# Supplementary material for: Antigen presentation plays positive roles in the regenerative response to cardiac injury in zebrafish
Source: Nat Commun. 2024 Apr 29;15:3637. doi: 10.1038/s41467-024-47430-1 (PMC11058276; doi:10.1038/s41467-024-47430-1)
Supplement: Supplementary file 1 — Supplementary Information [file 41467_2024_47430_MOESM1_ESM.pdf]

Supplementary Information for

Antigen presentation plays positive roles in the regenerative response to cardiac injury in zebrafish

João Cardeira-da-Silva[1,2,3,✉], Qianchen Wang[1,2,3], Pooja Sagvekar[1,2], Janita Mintcheva[4,5], Stephan Latting[1], Stefan Günther[2,3,6], Radhan Ramadass[1], Michail Yekelchyk[3,6], Jens Preussner[3,7], Mario Looso[2,3,7], Jan Philipp Junker[4,8,9], Didier Y. R. Stainier[1,2,3,✉]

[1] Department of Developmental Genetics, Max Planck Institute for Heart and Lung Research, Bad Nauheim, Germany
[2] DZHK German Centre for Cardiovascular Research, Partner Site Rhine-Main, Bad Nauheim, Germany
[3] Cardio-Pulmonary Institute (CPI), Bad Nauheim, Germany
[4] Max Delbrück Center for Molecular Medicine in the Helmholtz Association, Berlin Institute for Medical Systems Biology, Berlin, Germany
[5] Humboldt University of Berlin, Berlin, Germany
[6] Bioinformatics and Deep Sequencing Platform, Max Planck Institute for Heart and Lung Research, Bad Nauheim, Germany
[7] Bioinformatics Core Unit (BCU), Max Planck Institute for Heart and Lung Research, 61231 Bad Nauheim, Germany
[8] DZHK German Centre for Cardiovascular Research, Partner Site Berlin, Berlin, Germany
[9] Charité – Universitätsmedizin Berlin, Germany

✉: joao.cardeira-da-silva@mpi-bn.mpg.de; didier.stainier@mpi-bn.mpg.de

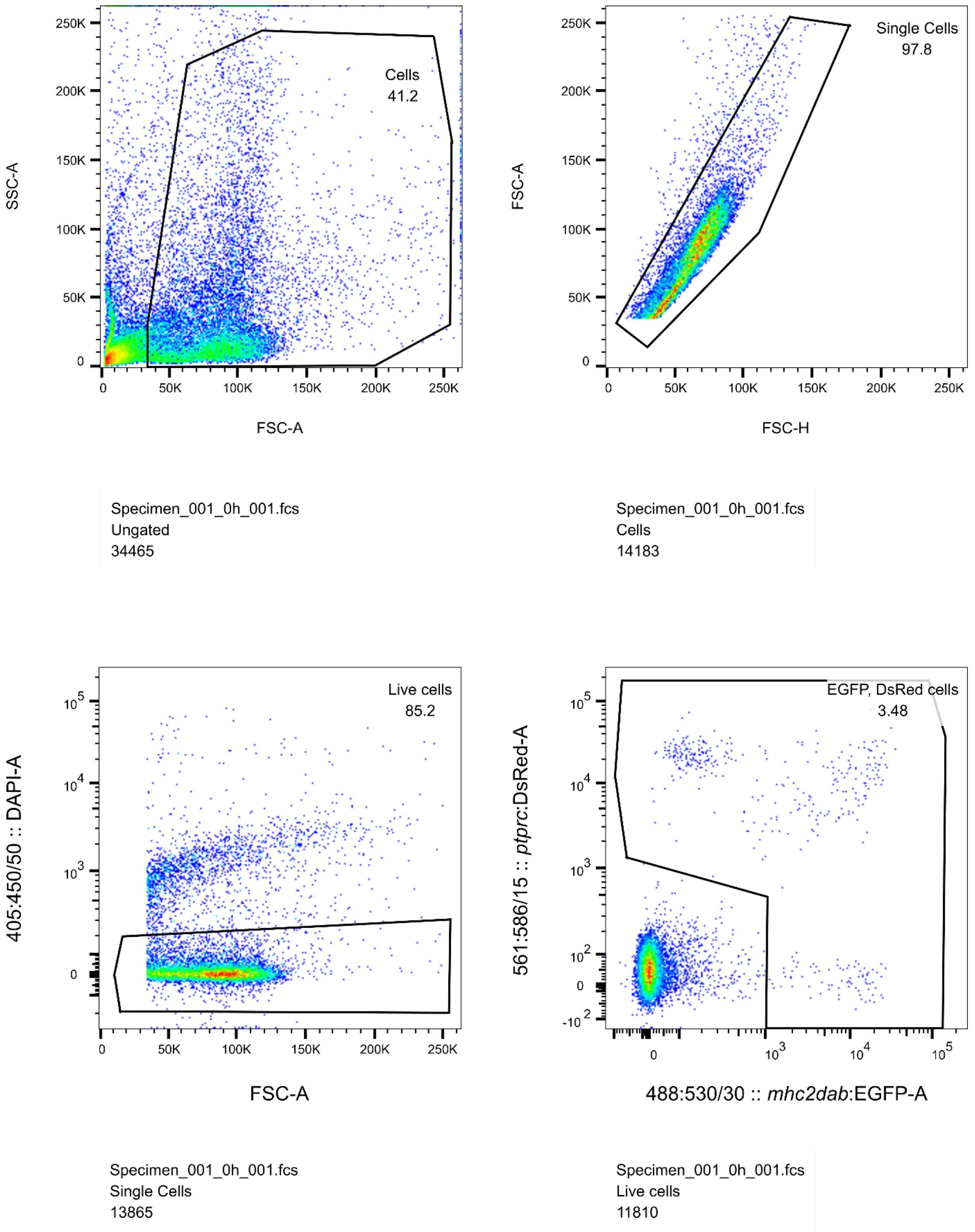

Supplementary Figure 1. Plots showing the hierarchical gating strategy for FACS-sorting live *Tg(mhc2dab:*EGFP*)*$^{+}$ and *Tg(ptprc:*DsRed*)*$^{+}$ (i.e., single and double positive) immune cells, excluding DAPI^{+} dead cells as well as non-fluorescent cells.

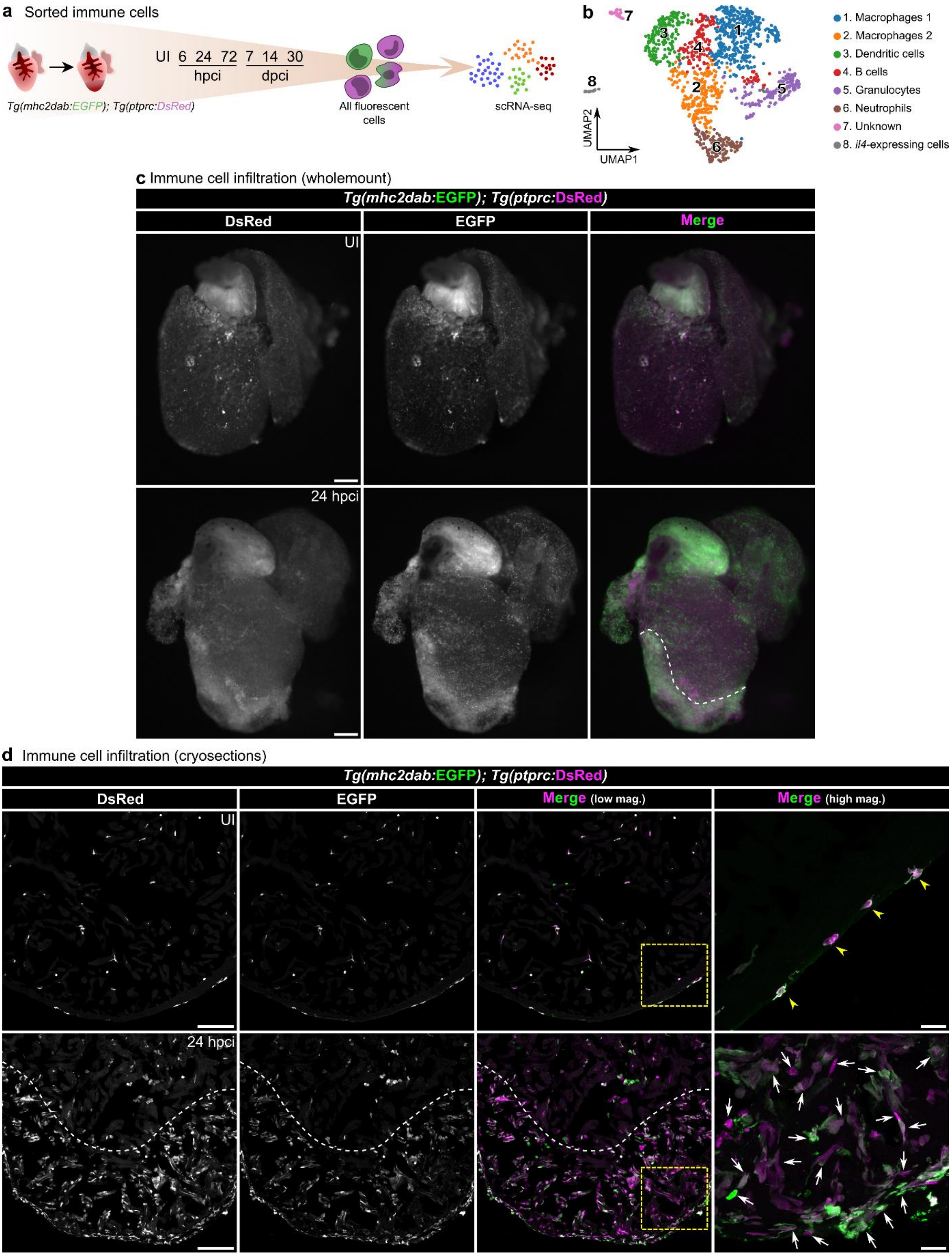

Supplementary Figure 2. **a, b** scRNA-Seq of sorted immune cells. **a** Experimental design for scRNA-Seq analysis of sorted immune cells from zebrafish hearts (i.e., uninjured, several time points post-cryoinjury, and a 72 hours post-sham sample). **b** UMAP plot of the clustering analysis of scRNA-Seq of sorted immune cells from all time points (including sham sample) reveals 8 major groups including APCs and lymphoid cells. **c** Images of representative wholemount hearts from adult *Tg(mhc2dab:GFP); Tg(ptprc:DsRed)* zebrafish showing resident (UI) and injury-infiltrating (24 hpci)

immune cells. **d** Images of representative cryosectioned uninjured and 24 hpci ventricles from adult *Tg(mhc2dab:GFP); Tg(ptprc:DsRed)* zebrafish, immunostained for GFP and DsRed and showing resident (yellow arrowheads; UI) and injury-infiltrating (white arrows; 24 hpci) immune cells in the adult heart; two independent experiments with similar results. Yellow dashed squares outline the magnified areas shown on the right; dashed lines mark the border of the injured tissue. Scale bars: 100 µm (low magnification); 20 µm (high magnification). UI, uninjured.

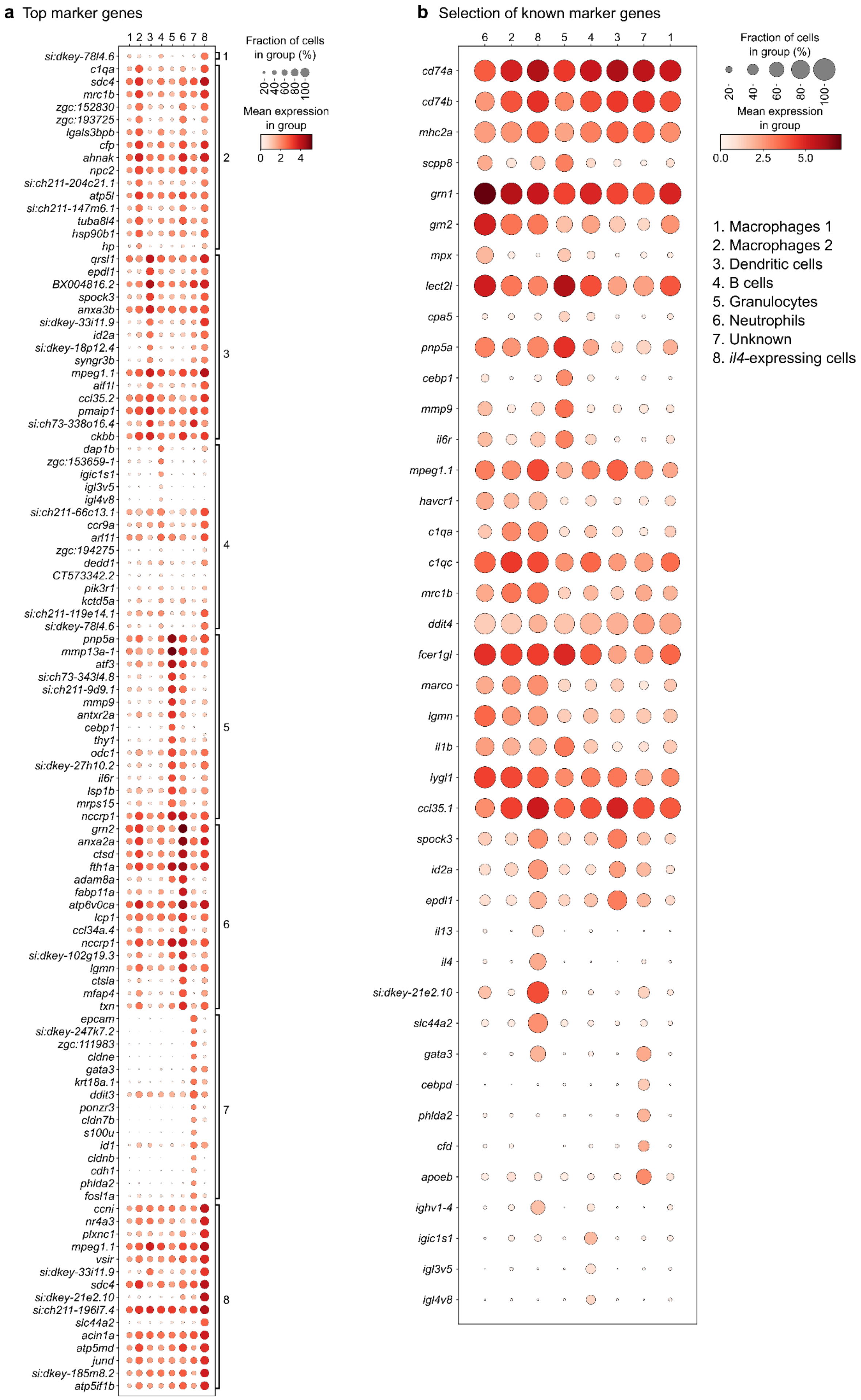
a Top marker genes
1 2 3 4 5 6 7 8
si:dkey-78l4.6
c1qa
sdc4
mrc1b
zgc:152830
zgc:193725
lgals3bpb
cfp
ahnak
npc2
si:ch211-204c21.1
atp5l
si:ch211-147m6.1
tuba8l4
hsp90b1
hp
qrsl1
epdl1
BX004816.2
spock3
anxa3b
si:dkey-33i11.9
id2a
si:dkey-18p12.4
syngr3b
mpeg1.1
aif1l
ccl35.2
pmaip1
si:ch73-338o16.4
ckbb
dap1b
zgc:153659-1
igic1s1
igl3v5
igl4v8
si:ch211-66c13.1
ccr9a
arl11
zgc:194275
dedd1
CT573342.2
pik3r1
kctd5a
si:ch211-119e14.1
si:dkey-78l4.6
pnp5a
mmp13a-1
atf3
si:ch73-343l4.8
si:ch211-9d9.1
mmp9
antxr2a
cebp1
thy1
odc1
si:dkey-27h10.2
il6r
lsp1b
mrps15
nccrp1
grn2
anxa2a
ctsd
fth1a
adam8a
fabp11a
atp6v0ca
lcp1
ccl34a.4
nccrp1
si:dkey-102g19.3
lgmn
ctsla
mfap4
txn
epcam
si:dkey-247k7.2
zgc:111983
cldne
gata3
krt18a.1
ddit3
ponzr3
cldn7b
s100u
id1
cldnb
cdh1
phlda2
fosl1a
ccni
nr4a3
plxnc1
mpeg1.1
vsir
si:dkey-33i11.9
sdc4
si:dkey-21e2.10
si:ch211-196l7.4
slc44a2
acin1a
atp5md
jund
si:dkey-185m8.2
atp5if1b
1
2
3
4
5
6
7
8
Fraction of cells in group (%)
20 40 60 80 100
Mean expression in group
0 2 4
b Selection of known marker genes
6 2 8 5 4 3 7 1
cd74a
cd74b
mhc2a
scpp8
grn1
grn2
mpx
lect2l
cpa5
pnp5a
cebp1
mmp9
il6r
mpeg1.1
havcr1
c1qa
c1qc
mrc1b
ddit4
fcer1gl
marco
lgmn
il1b
lygl1
ccl35.1
spock3
id2a
epdl1
il13
il4
si:dkey-21e2.10
slc44a2
gata3
cebpd
phlda2
cfd
apoeb
ighv1-4
igic1s1
igl3v5
igl4v8
Fraction of cells in group (%)
20 40 60 80 100
Mean expression in group
0.0 2.5 5.0
1. Macrophages 1
2. Macrophages 2
3. Dendritic cells
4. B cells
5. Granulocytes
6. Neutrophils
7. Unknown
8. il4-expressing cells

Supplementary Figure 3. a, b Dot plots showing the expression of top marker genes (**a**) and of a selection of known marker genes of different immune subsets (**b**), across the various clusters.

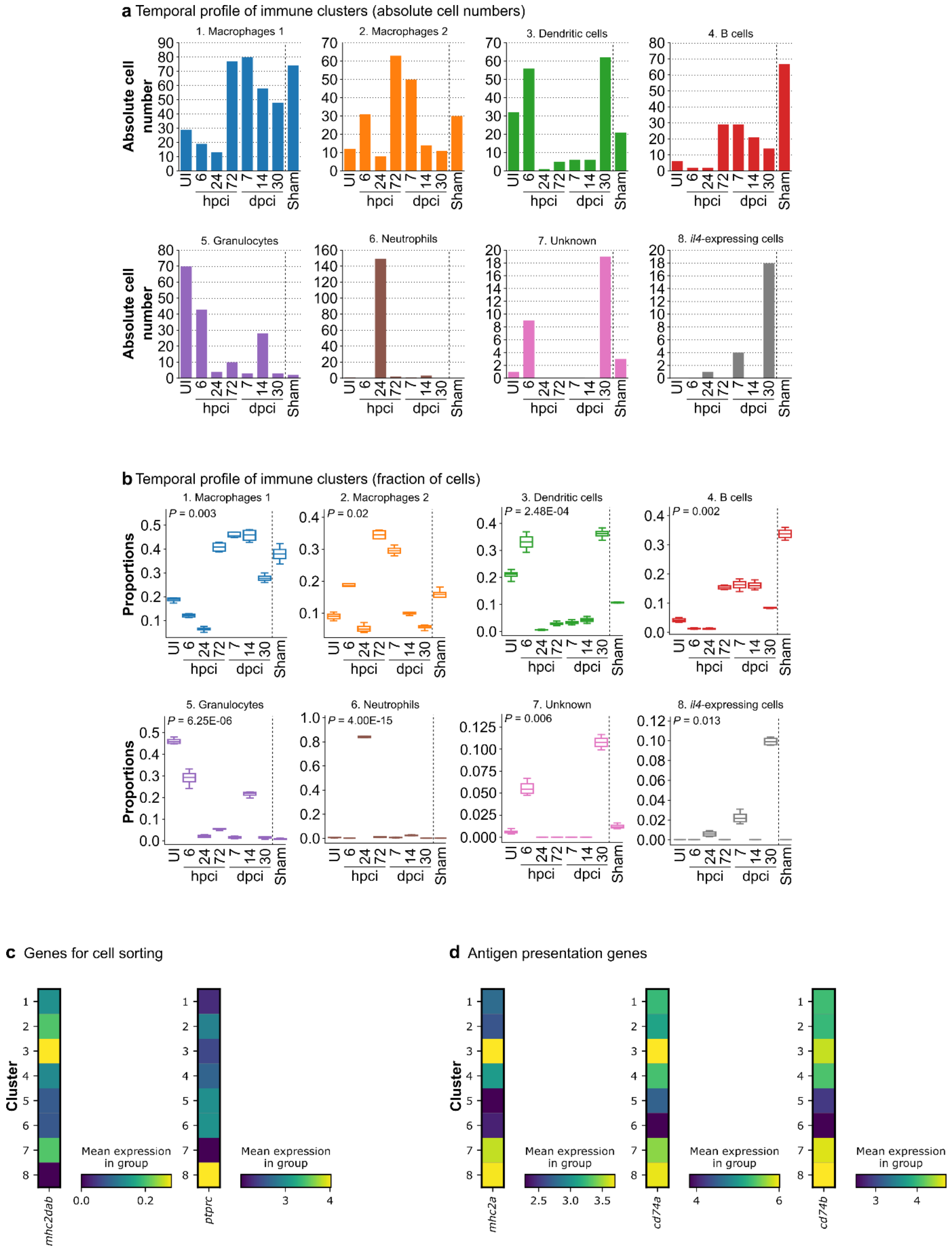

Supplementary Figure 4. a, b Bar and box plots showing absolute cell numbers (**a**) and proportion (**b**) of the various clusters at the different time points and conditions. Sham sample refers to 72 hours post-sham. Box plots represent the median, Q1, Q3, the minimum, and the maximum; one-way ANOVA (*P* values included in the graphs). **c, d** Heat maps showing the expression of the genes whose regulatory elements were used to FACS-sort the immune cells (**c**) and of MHC class II antigen presentation genes (**d**) across the various immune cell clusters.

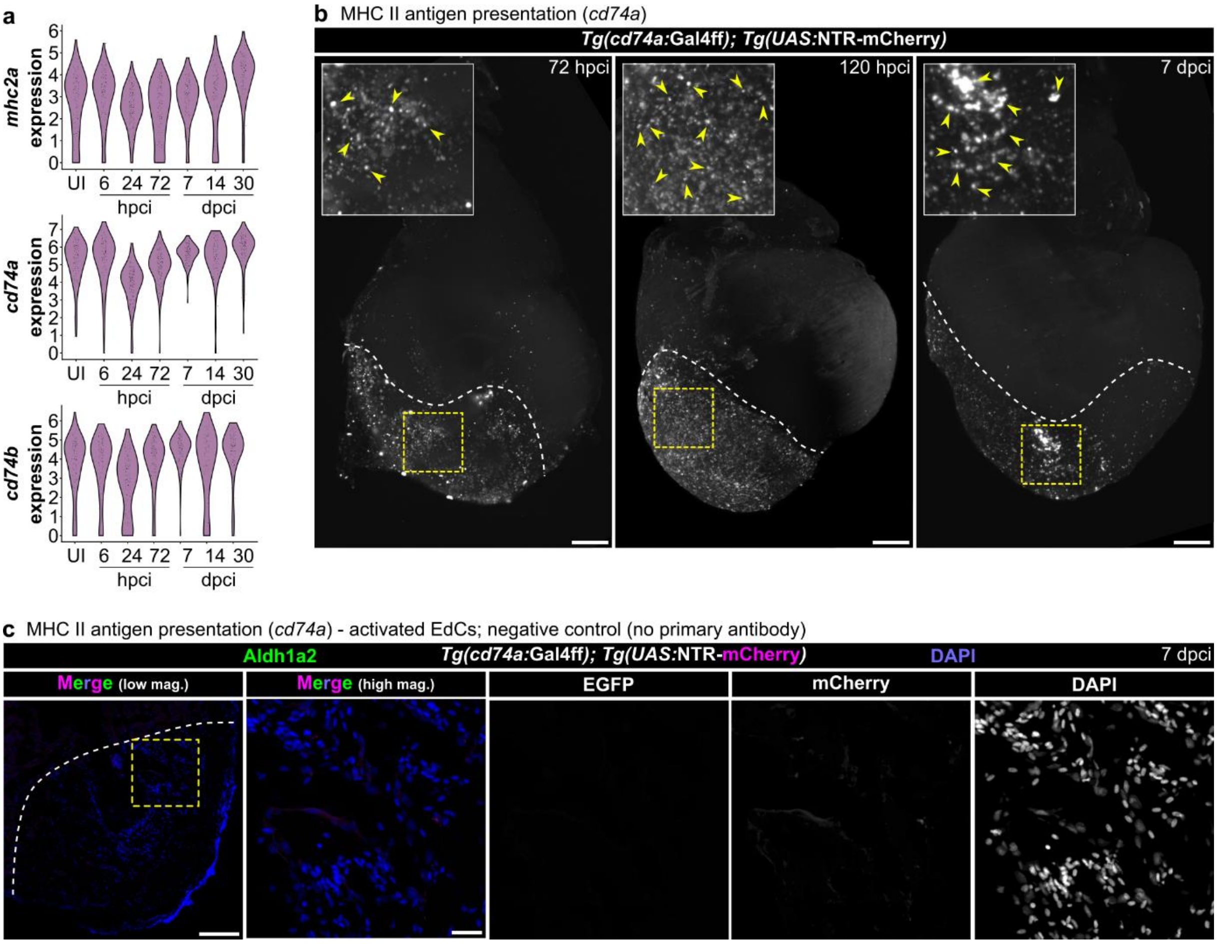

Supplementary Figure 5. **a** Violin plots showing the expression of MHC class II antigen presentation genes over time in all cells from the scRNA-Seq data of sorted immune cells, revealing an initial decrease followed by an increase starting at 72 hpci. **b** Lightsheet images of representative wholemount ventricles from adult *Tg(cd74a:Gal4ff); Tg(UAS:NTR-mCherry)* zebrafish at 72 and 120 hpci, and at 7 dpci, showing mCherry^{+} cells (yellow arrowheads); two independent experiments with similar results. **c** Confocal images of a cryosectioned ventricle from an adult *Tg(cd74a:Gal4ff); Tg(UAS:NTR-mCherry)* zebrafish at 120 hpci, as a negative control for the immunostaining for mCherry and Aldh1a2 (Fig. 1c); one experiment. Yellow dashed squares outline the magnified areas; dashed lines mark the border of the injured tissue. Scale bars: 100 µm.

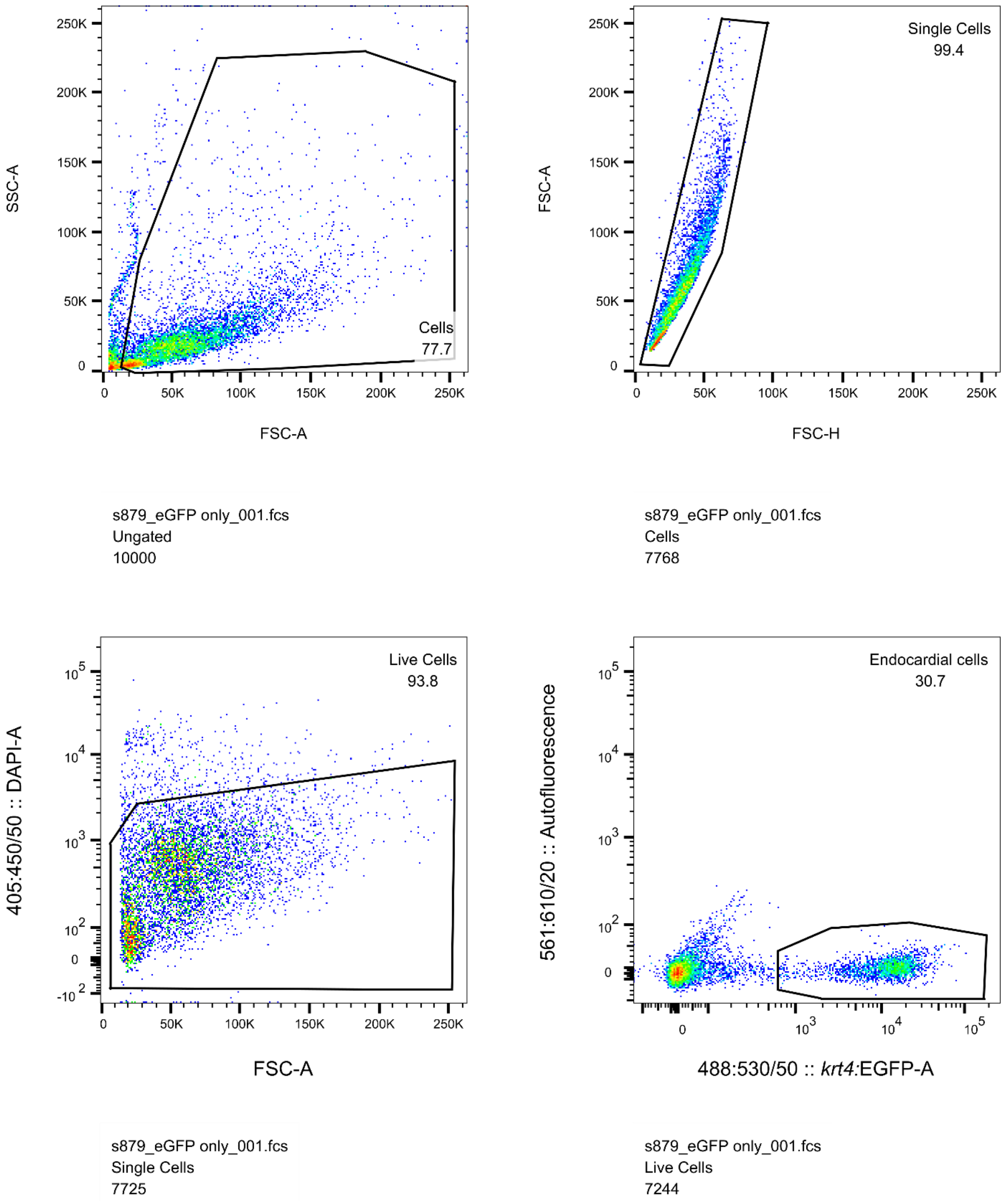

Supplementary Figure 6. Plots showing the hierarchical gating strategy for FACS-sorting live *Et(krt4:*EGFP*)*$^{+}$ EdCs, excluding DAPI^{+} dead cells as well as non-fluorescent cells.

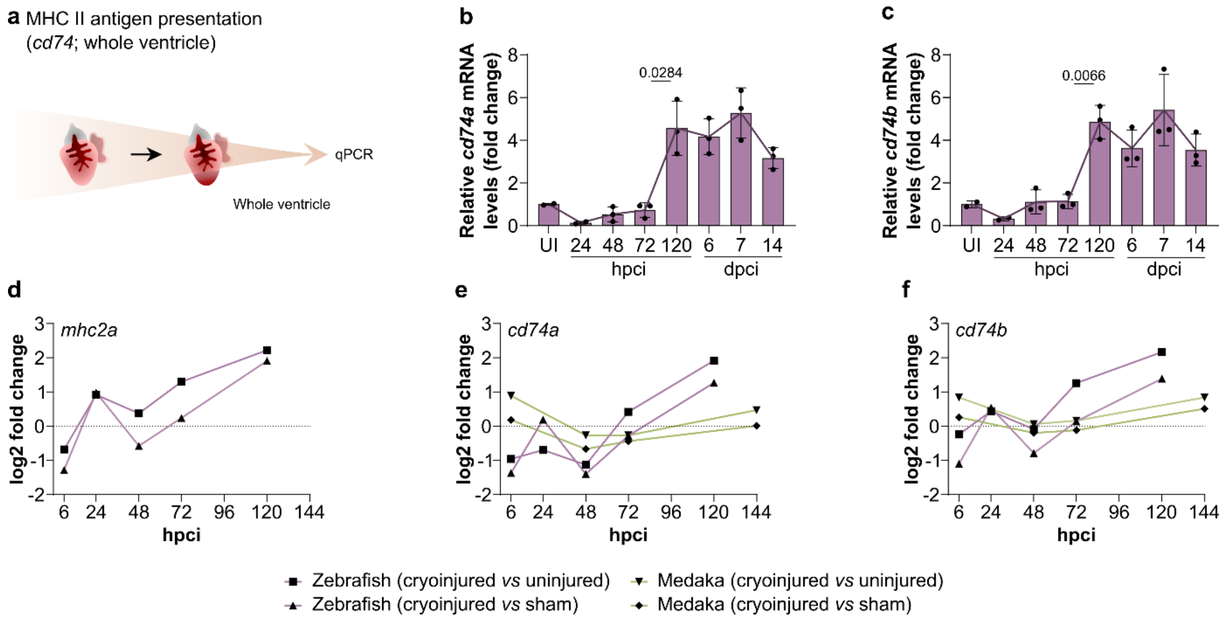

Supplementary Figure 7. a-c qPCR analyses of whole ventricles (**a**) showing the relative mRNA levels of *cd74a* (**b**) and *cd74b* (**c**) at various time points post-cryoinjury, showing increased expression starting at 120 hpci. Dots in the graphs represent individual ventricles and the mean ± SD is represented; n = 2 biologically independent samples for UI and 24 hpci, and 3 biologically independent samples for the remaining time points; two-tailed Welch's *t* test between 72 and 120 hpci (*P* values included in the graphs); Ct values included in Supplementary Table 10. **d-f** Graphs showing increased expression of *mhc2a* (**d**), *cd74a* (**e**), and *cd74b* (**f**) in cryoinjured zebrafish hearts starting at 72 hpci but not in medaka hearts, at least for *cd74a* and *cd74b*; medaka ortholog(s) of zebrafish *mhc2a* has/have not yet been assigned. Data extracted from published RNA-seq dataset[30].

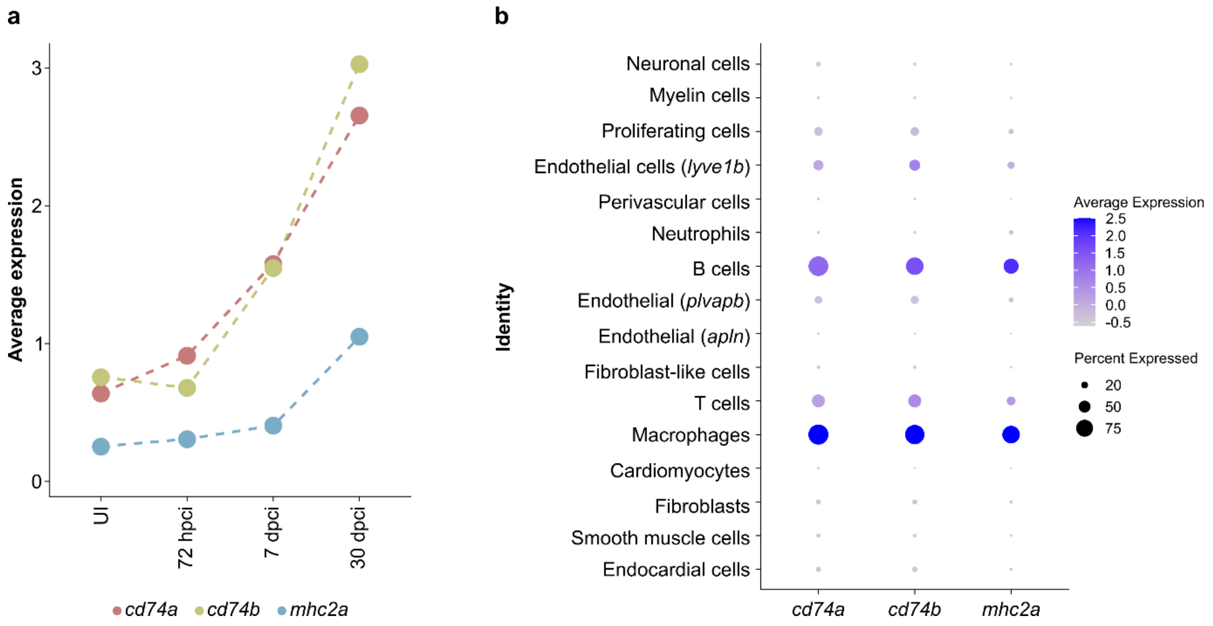

Supplementary Figure 8. **a** Plot showing the average expression of antigen presentation genes within the whole EdC population of the zebrafish heart at different time points after cryoinjury, revealing an increase during regeneration. **b** Dot plot showing the expression of antigen presentation genes across the major cell groups. Data extracted from published scRNA-seq dataset[43].

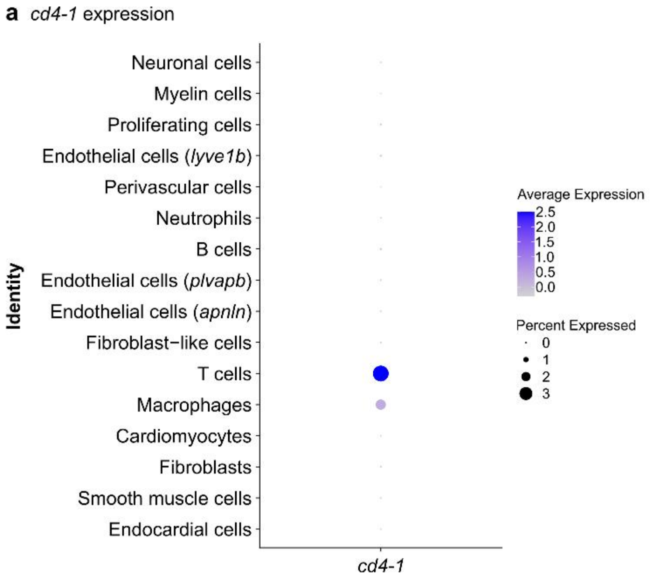

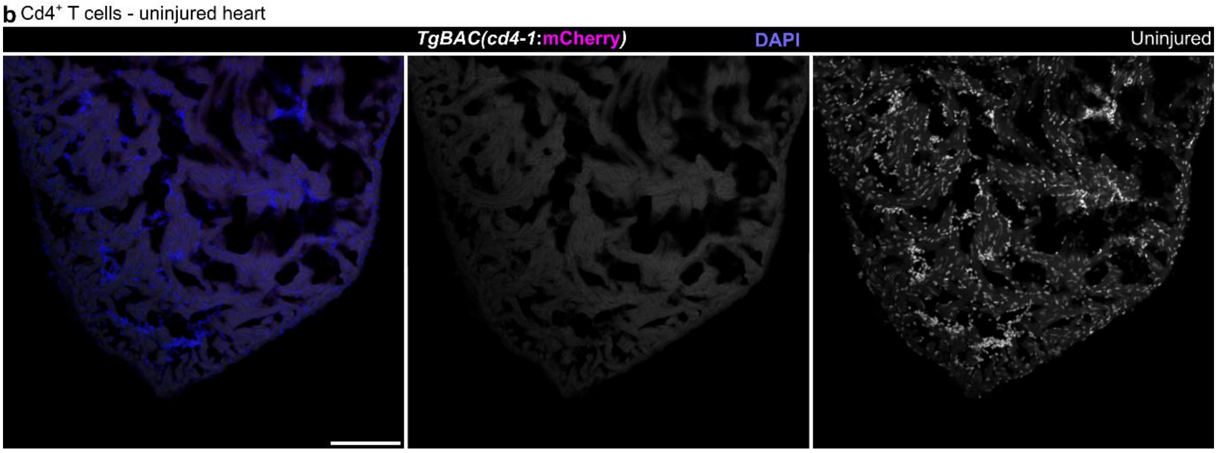

Supplementary Figure 9. **a** Dot plot of *cd4-1* expression across the various cell types in the regenerating zebrafish heart, showing high specificity to T cells. Data extracted from published scRNA-seq dataset[43]. **b** Confocal images of a representative uninjured ventricle from an adult *TgBAC(cd4-1:mCherry)* zebrafish, immunostained for mCherry, showing no Cd4^{+} T cells; two independent experiments with similar results. Scale bar: 100 µm.

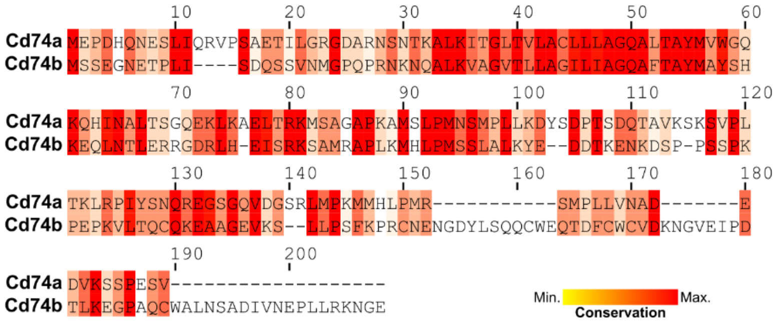

Supplementary Figure 10. Alignment of the zebrafish Cd74a and Cd74b sequences, showing high homology.

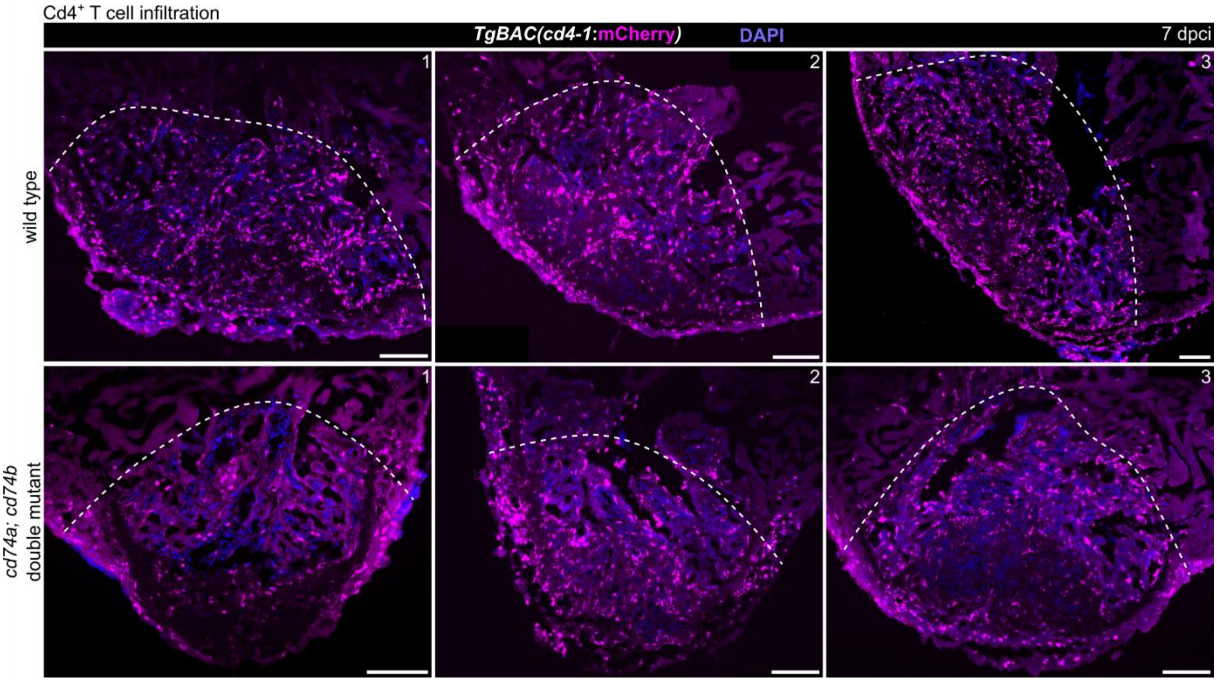

Supplementary Figure 11. Additional confocal images of representative cryosectioned ventricles from adult *Tg(cd4-1:mCherry)* wild-type and *cd74a; cd74b* mutant zebrafish at 7 dpci, immunostained for mCherry. Dashed lines mark the border of the injured tissue. Scale bars: 100 µm.

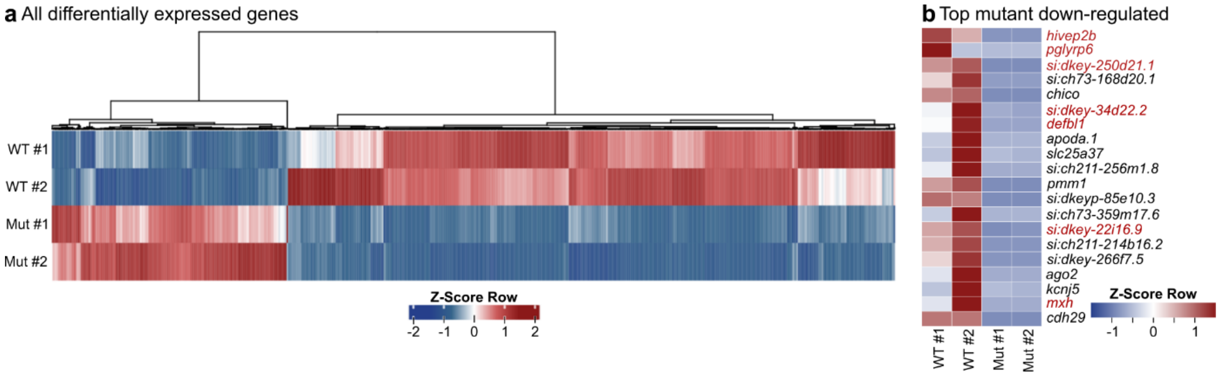

Supplementary Figure 12. a, b Heat maps showing the relative expression of all differentially expressed genes (**a**) and the top 20 genes down-regulated in *cd74a; cd74b* mutants (**b**) from bulk RNA-Seq of zebrafish ventricles at 120 hpci (from Fig. 5). Genes with known or predicted immune functions in **b** are marked in red. WT, wild-type samples; Mut, *cd74a; cd74b* mutant samples.

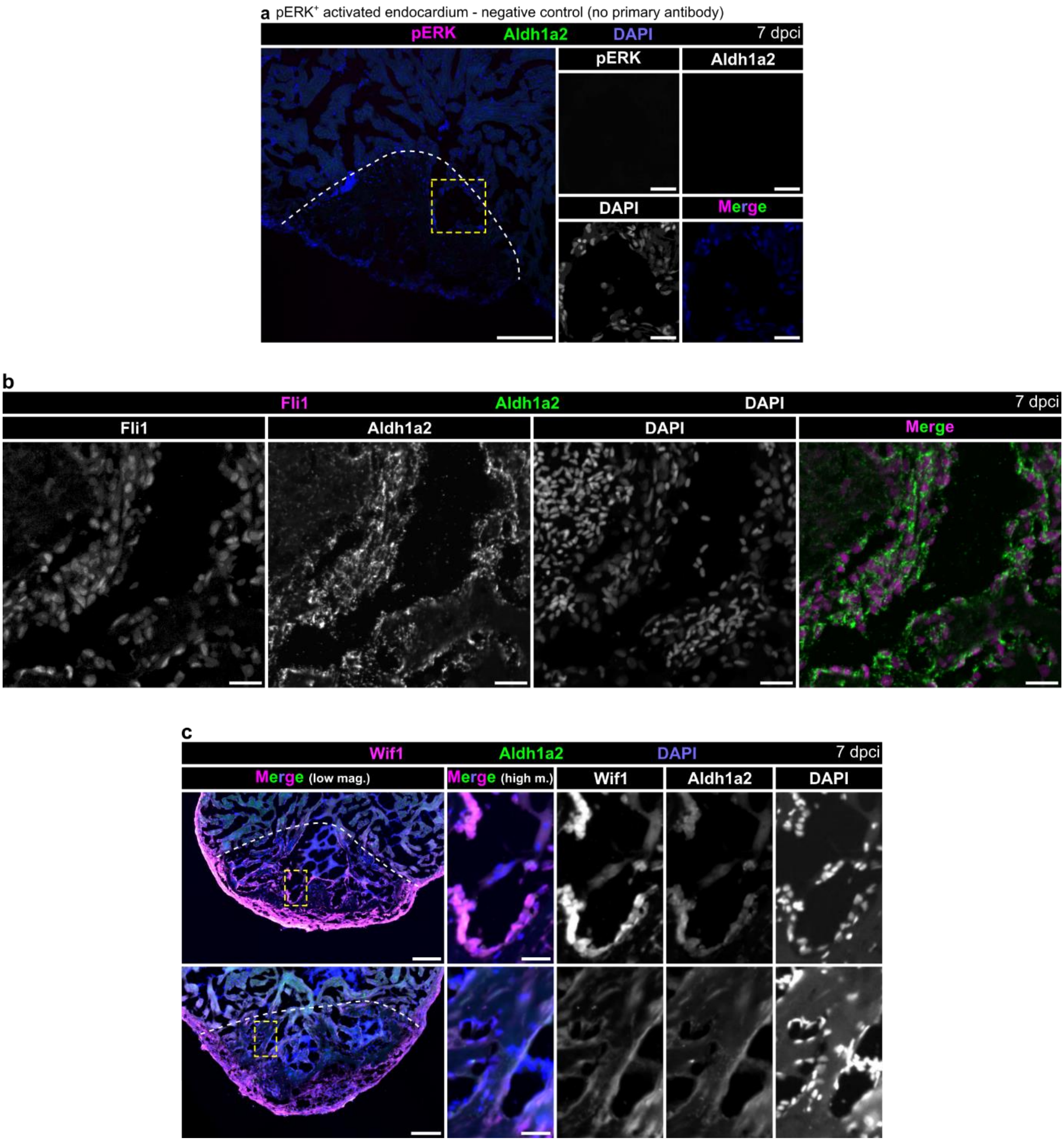

Supplementary Figure 13. **a** Confocal images of a cryosectioned adult zebrafish ventricle at 7 dpci, as a negative control for the immunostaining for pERK and Aldh1a2 (Fig. 6a); one experiment. **b** Confocal images of a representative cryosectioned ventricle (injured area) from an adult zebrafish at 7 dpci immunostained for Fli1 and Aldh1a2 and showing that both signals localize to the same cells; two independent experiments with similar results. **c** Images of representative cryosectioned ventricles from adult zebrafish at 7 dpci, immunostained for Wif1 and Aldh1a2; one experiment. Yellow dashed squares outline the magnified areas; dashed lines mark the border of the injured tissue. Scale bars: 100 µm in **a** and **c** (low mag); 20 µm in **a-c** (high mag.).

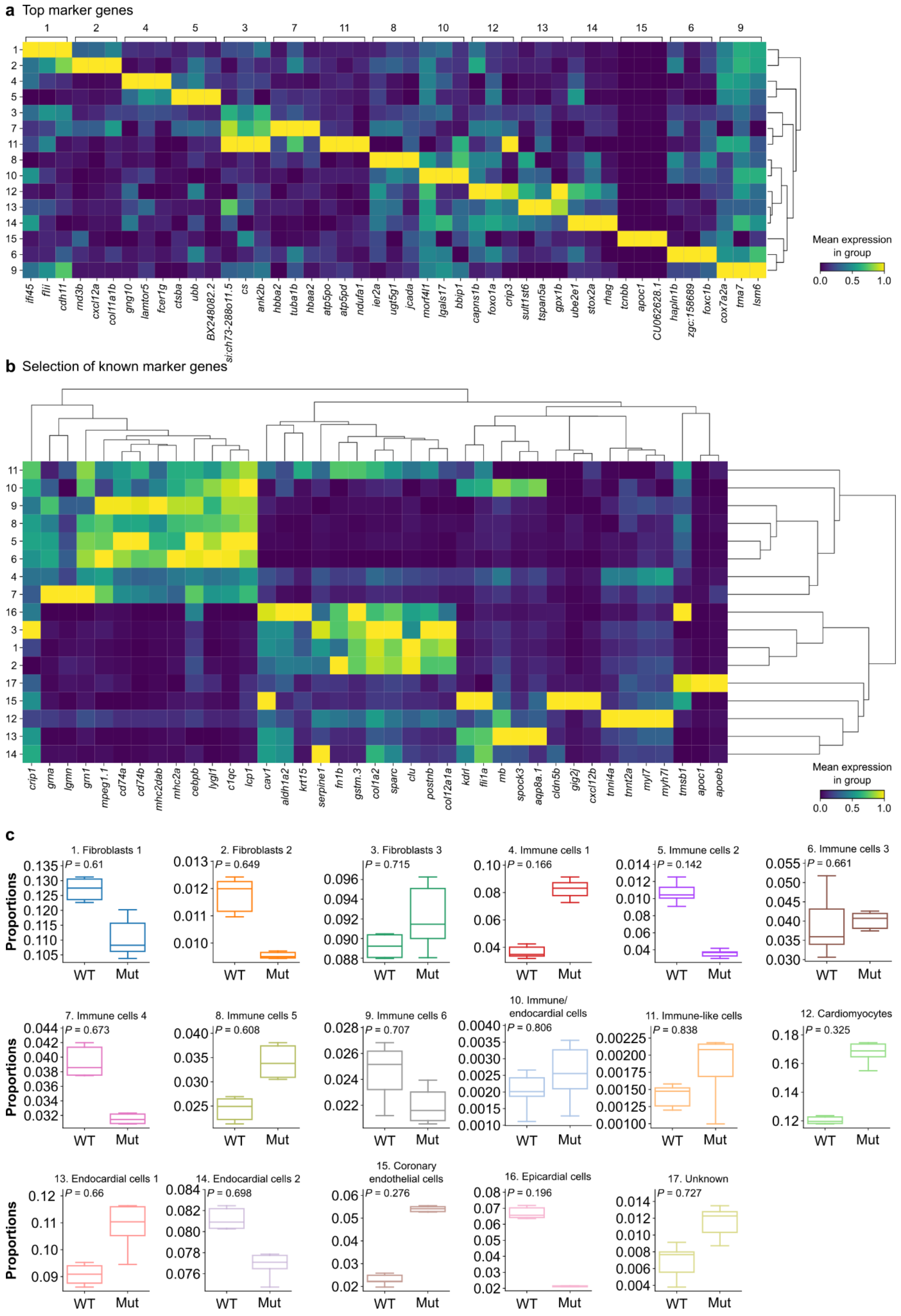

Supplementary Figure 14. a, b Matrix plots showing the relative expression of top marker genes (**a**) and a selection of known marker genes (**b**) per cluster; data extracted from the scRNA-seq dataset of

injured and border zone tissues of wild types versus *cd74a; cd74b* mutants at 120 hpci. Rows in **a** and **b** represent the different clusters; numbers on columns in **a** indicate the top three marker genes per cluster. **c** Box plots showing the proportion of the various clusters in wild types (WT) and *cd74a; cd74b* mutants (Mut). Box plots represent the median, Q1, Q3, the minimum, and the maximum; student's *t* test (*P* values included in the graph).

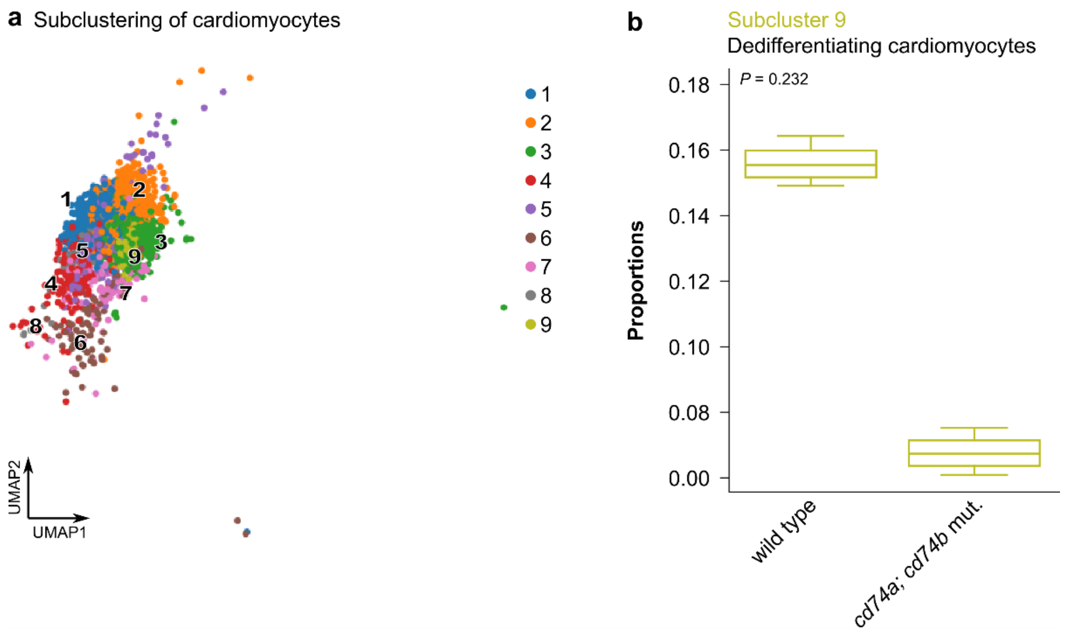

Supplementary Figure 15. **a** Subclustering of cardiomyocytes from the scRNA-seq dataset injured and border zone tissues of wild types versus *cd74a; cd74b* mutants (Fig. 6h,i). **b** Box plot showing the proportion of subcluster 9 in wild types and *cd74a; cd74b* mutants. Box plots represent the median, Q1, Q3, the minimum, and the maximum; student's *t* test (*P* values included in the graph).

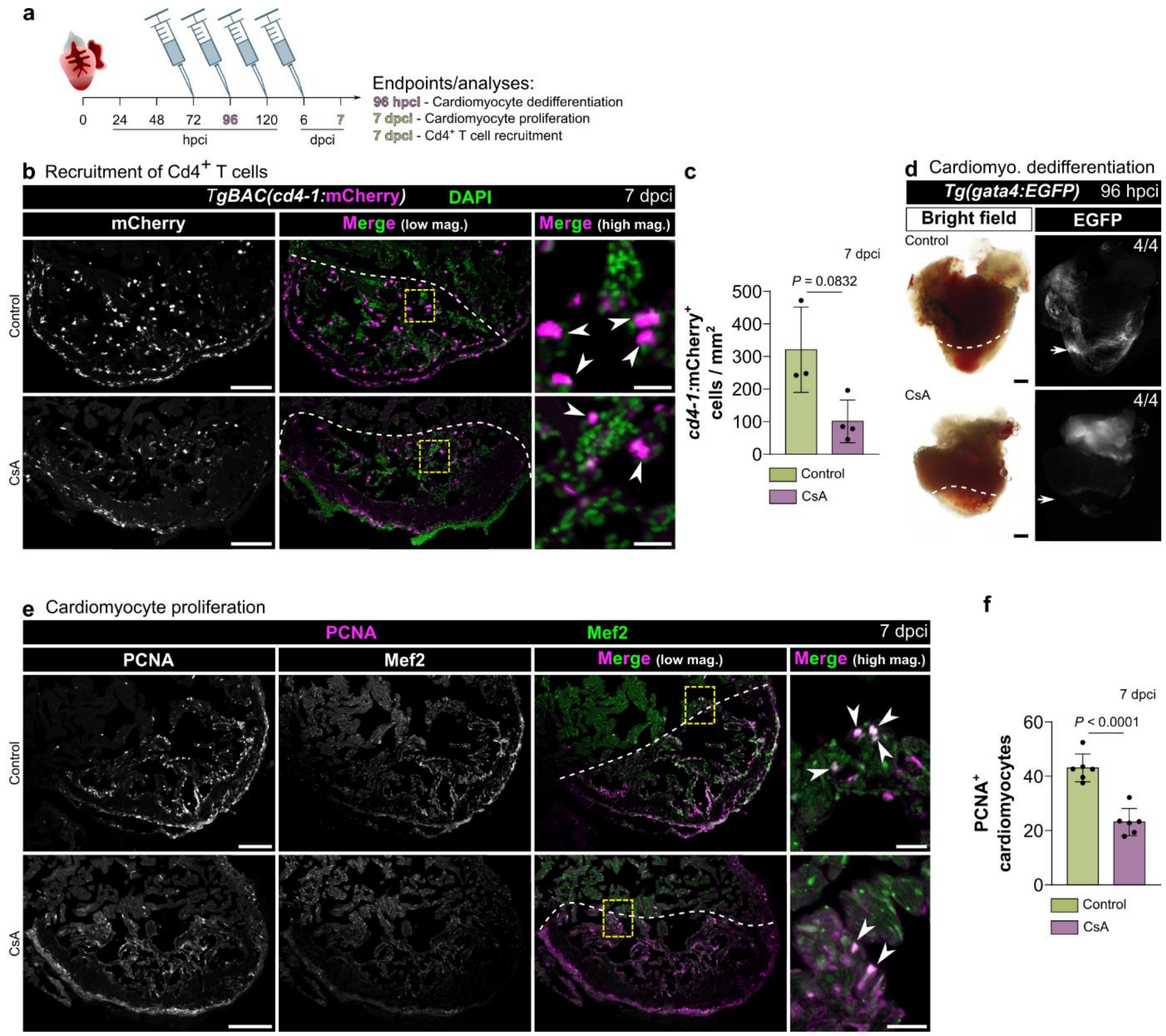

Supplementary Figure 16. Cyclosporine A treatment results in impaired cardiomyocyte dedifferentiation and proliferation. **a** CsA treatment protocol, indicating daily intraperitoneal injections from 72 hpci to 6 dpci. **b, c** Confocal images of representative cryosectioned ventricles from adult zebrafish at 7 dpci (**b**) and quantification (**c**) of *cd4-1:*mCherry^{+} T cells (arrowheads in **b**), showing a tendency for decreased numbers in CsA-treated compared with control zebrafish at this stage. **d** Stereomicroscope images of representative wholemount hearts from adult *Tg(gata4:EGFP)* zebrafish at 96 hpci, displaying lack of expression in the injury border zone (white arrows) of CsA-treated compared with control zebrafish; one experiment. **e, f** Confocal images of representative cryosectioned ventricles from adult zebrafish at 7 dpci, immunostained for Mef2 and PCNA (**e**) and quantification of proliferating cardiomyocytes (**f**; arrowheads in **e**), showing a decrease in CsA-treated compared with control zebrafish. Dots in the bar graphs represent individual ventricles, and the bars represent the mean ± SD; n = 3 (Control) and 4 (CsA) biologically independent samples in **c**, and 6 (Control and CsA) biologically independent samples in **f**; two-tailed Welch's *t* test (*P* values included in the graphs). Yellow dashed squares and rectangles outline the magnified areas; dashed lines mark the border of the injured tissue. Scale bars: 100 µm (low magnification); 20 µm (high magnification).

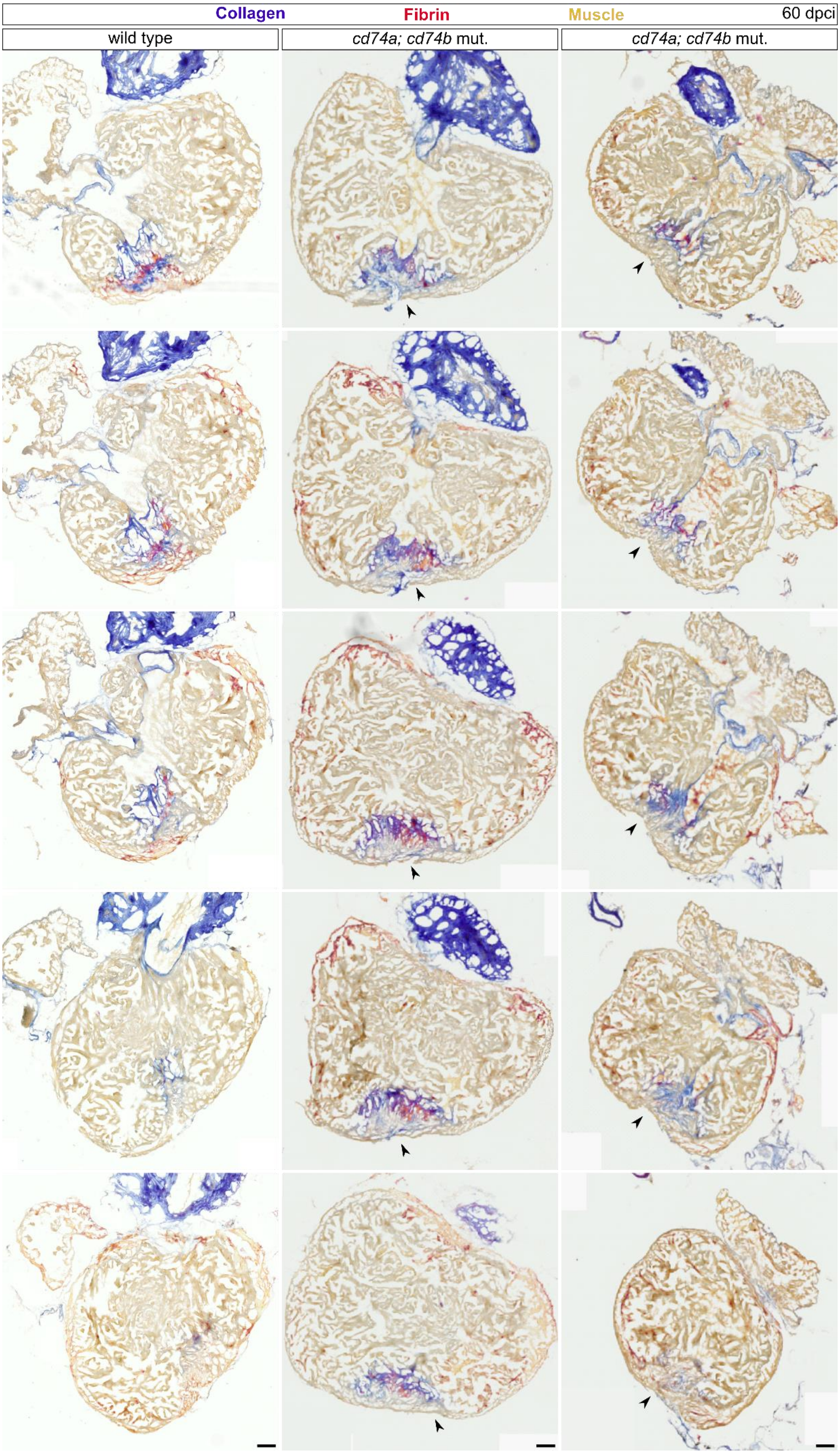
Collagen
Fibrin
Muscle
60 dpci
wild type
cd74a; cd74b mut.
cd74a; cd74b mut.

Supplementary Figure 17. Brightfield images of serial AFOG-stained cryosections of one representative wild-type and two representative *cd74a; cd74b* mutant ventricles from adult zebrafish at 60 dpci. Arrowheads point to tissue constrictions close to the scar. Collagen, blue; fibrin, red; muscle, orange. Scale bars: 100 µm.

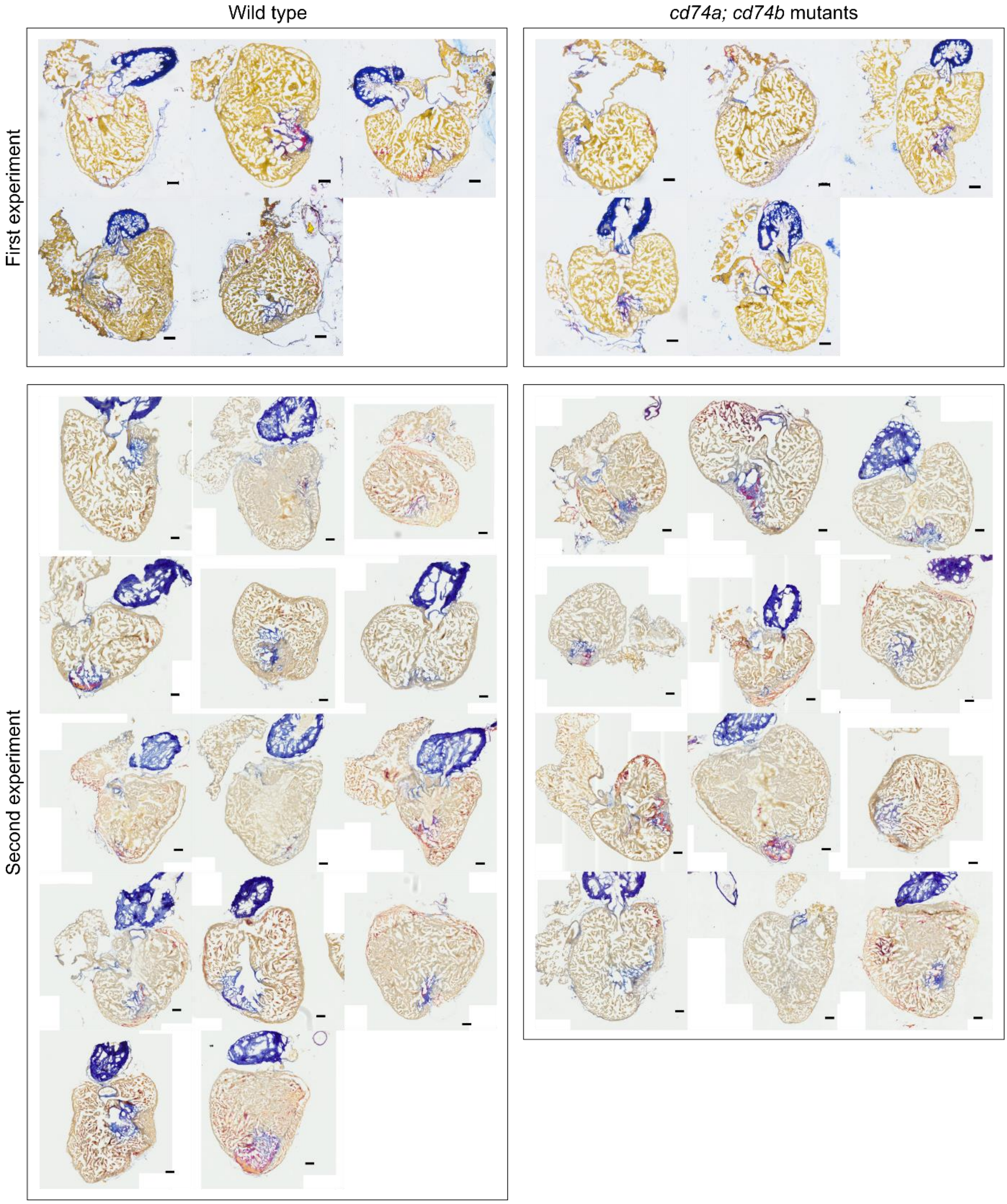

Supplementary Figure 18. Brightfield images of AFOG-stained cryosections from all the wild-type and *cd74a; cd74b* mutant ventricles from adult zebrafish at 60 dpci. The scar tissue is revealed by the collagen staining (blue). Collagen, blue; fibrin, red; muscle, orange. Scale bars: 100 µm

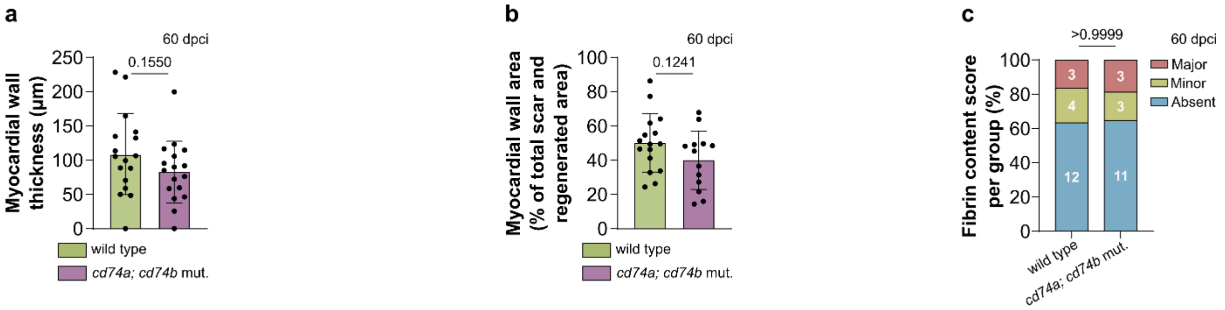

Supplementary Figure 19. a, b Quantification of myocardial wall thickness (**a**) and myocardial wall area (**b**) at the injury site in AFOG-stained cryosections from wild-type and *cd74a; cd74b* mutant ventricles from adult zebrafish at 60 dpci. Dots in the graphs represent individual ventricles, and the bars represent the mean ± SD; n = 17 (wild type and *cd74a; cd74b* mut.) biologically independent samples in **a**, and 16 (wild type) and 13 (*cd74a; cd74b* mut.) biologically independent samples in **b**. **c,** Fibrin content index in wild-type and *cd74a; cd74b* mutant ventricles from adult zebrafish at 60 dpci. White numbers represent the counts per category. Two-tailed Welch's *t* test in **a** and **b**, and Fisher's exact test in **c** (*P* values included in the graphs).

Supplementary Table 1. Total number of cells per condition and per cluster from scRNA-Seq data of sorted immune cells; related to Supplementary Figs. 2-4. UI, uninjured.

Condition	Number of cells
UI	151
6 hpci	160
24 hpci	178
72 hpci	186
72 hps (sham)	197
7 dpci	173
14 dpci	130
30 dpci	175
Total	***1350***

Cluster	Number of cells
1	398
2	219
3	189
4	170
5	163
6	156
7	32
8	23
**Total**	***1350***

Supplementary Table 2. Normalized expression of *Cd74* in endocardial cells from sham hearts and hearts post-myocardial infarction (MI) from P1 and P8 mice. Average expression values (counts per 10,000) from both day 1 and 3 combined post-sham and post-MI. Data extracted from published scRNA-Seq dataset[42].

Gene	P1 sham	P1 MI	P8 sham	P8 MI
Cd74	0	0.3776	0.0797	0

Supplementary Table 3. Full list of multiple comparisons of *cd4-1:*mCherry⁺ T cell accumulation in the injured tissue at various time points; related to Fig. 3b. One-way ANOVA (P = 0.0003) and Tukey's post hoc test for multiple comparisons. ns, non-significant.

Tukey's multiple comparisons test	Summary	Adjusted P Value
24 hpci vs. 72 hpci	ns	0.9674
24 hpci vs. 96 hpci	ns	0.8715
24 hpci vs. 120 dpci	ns	0.3351
24 hpci vs. 6 dpci	ns	0.1601
24 hpci vs. 7 dpci	***	0.0003
24 hpci vs. 14 dpci	ns	0.7667
24 hpci vs. 30 dpci	ns	0.9997
72 hpci vs. 96 hpci	ns	>0.9999
72 hpci vs. 120 dpci	ns	0.9303
72 hpci vs. 6 dpci	ns	0.6607
72 hpci vs. 7 dpci	**	0.0044
72 hpci vs. 14 dpci	ns	0.9996
72 hpci vs. 30 dpci	ns	0.9992
96 hpci vs. 120 dpci	ns	0.9910
96 hpci vs. 6 dpci	ns	0.8419
96 hpci vs. 7 dpci	*	0.0101
96 hpci vs. 14 dpci	ns	>0.9999
96 hpci vs. 30 dpci	ns	0.9851
120 dpci vs. 6 dpci	ns	0.9928
120 dpci vs. 7 dpci	*	0.0194
120 dpci vs. 14 dpci	ns	0.9948
120 dpci vs. 30 dpci	ns	0.6364
6 dpci vs. 7 dpci	ns	0.2429
6 dpci vs. 14 dpci	ns	0.8539
6 dpci vs. 30 dpci	ns	0.3440
7 dpci vs. 14 dpci	**	0.0062
7 dpci vs. 30 dpci	**	0.0011
14 dpci vs. 30 dpci	ns	0.9581

Supplementary Table 4. Total number of cells per cluster from scRNA-Seq data of wild-type versus *cd74a*; *cd74b* mutant ventricular tissue; related to Fig. 6g,h.

Cluster	Number of cells
1	1230
2	1140
3	971
4	742
5	626
6	414
7	385
8	342
9	258
10	32
11	23
12	1691
13	1095
14	876
15	482
16	396
17	124
Total	***10827***

Supplementary Table 5. Normalized cell fraction of the various cardiomyocyte subclusters in wild-type and *cd74a*; *cd74b* mutant ventricular tissue; related to Supplementary Fig. 15.

	**Cluster**								
	**1**	**2**	**3**	**4**	**5**	**6**	**7**	**8**	**9**
**wild type**	0.2482	0.1522	0.1148	0.1475	0.0468	0.0796	0.0304	0.0234	0.1569
***cd74a; cd74b* mutant**	0.2089	0.2389	0.2168	0.0759	0.0665	0.0514	0.0506	0.0245	0.0665

Supplementary Table 6. Average Ct values of *mhc2a*, *cd74a*, *cd74b* and *rpl13* expression per time point; related to Fig. 1f-h.

	mhc2a	***cd74a***	***cd74b***	***rpl13***
UI	34.34	33.94	34.43	28.85
24 hpci	N/A	36.51	35.95	28.41
120 hpci	32.40	31.47	32.07	27.82
7 dpci	30.50	29.41	30.56	26.14
14 dpci	30.81	29.21	29.99	27.75

Supplementary Table 7. Average Ct values of *cd74a*, *cd74b*, *rag2, mhc2a*, and *rpl13* expression at 72 hpci and 7 dpci; related to Fig. 4d-g. WT, wild types; Mut, *cd74a; cd74b* mutants.

Experiment 1		***cd74a***		***cd74b***		***rag2***		***rpl13***	
		WT	Mut	WT	Mut	WT	Mut	WT	Mut
	72 hpci	23.35	25.7	22.98	27.4	31.78	34.66	19.96	20.18
	7 dpci	22.67	27.16	23.61	29.34	33.17	35.74	20.26	20.68

Experiment 2		***mhc2a***		***rpl13***	
		WT	Mut	WT	Mut
	72 hpci	23.44	22.74	20.96	20.46
	7 dpci	24.65	25.13	21.71	21.98

Supplementary Table 8. List of qPCR primers.

	Forward primer (5'-3')	Reverse primer (5'-3')	Note
cd74a	ACCAGGAAGATGTCAGCTGGAGC	TTGACGGCGGTCTGGTCAGAAG	(1)
	GTCGAGGTGATGCAAGGAACAGC	CTCAGCCTTGAGTTTCTCCTGTCC	(2)
cd74b	AGATCAGCCGTAAATCTGCCATGCG	GCTCTGGTTTGGGAGATGAGGGA	(1)
	ACATGGGACCTCAGCCAAGAAATAAG	TTATGACTGTAGGCCATGTAAGCAGTG	(2)
rag2	CAACCTTCACAAACACTCAACC	TTTGAAGGTAGCTCTTGGTCAG	
mhc2a	GGCACCTTCAACATGTTCTCTGCTC	AGCTCAACGTCCACCTCCCATG	
rpl13	TAAGGACGGAGTGAACAACCA	CTTACGTCTGCGGATCTTTCTG	

(1) To determine mRNA levels in cd74a; cd74b mutants (Fig. 4d,e)
(2) All other reactions

Supplementary Table 9. Average Ct values of *cd74a*, *cd74b*, and *rpl13* expression per time point; related to Supplementary Fig. 7b,c.

	cd74a	***cd74b***	***rpl13***
UI	23.63	25.87	20.89
24 hpci	26.71	27.67	21.05
48 hpci	24.68	25.7	20.78
72 hpci	24.38	25.88	21.06
120 hpci	21.62	23.74	21.04
6 dpci	21.36	23.79	20.67
7 dpci	20.93	23.14	20.57
14 dpci	22.73	24.81	21.64
