# Supplementary material for: Antigen presentation plays positive roles in the regenerative response to cardiac injury in zebrafish
Source: Nat Commun. 2024 Apr 29;15:3637. doi: 10.1038/s41467-024-47430-1 (PMC11058276; doi:10.1038/s41467-024-47430-1)
Supplement: Supplementary file 3 — Description of Additional Supplementary Files [file 41467_2024_47430_MOESM3_ESM.pdf]

Description of Additional Supplementary Files:

Supplementary Data 1: Pairwise comparison of cd74a, cd74b and mhc2a mRNA levels across clusters from sorted immune cells. Related to Supplementary Fig. 4b. Mean expression, fold changes, P values and adjusted P values are included.

Supplementary Data 2: Top marker genes per endocardial subcluster from published dataset43 . Related to Fig. 2.

Supplementary Data 3: List of differentially expressed genes between wild-type and cd74a; cd74b mutant ventricular tissue within endocardial subclusters 13 and 14. Related to Fig. 6i,j. Statistics are included.

Supplementary Data 4: List of differentially expressed genes between wild-type and cd74a; cd74b mutant ventricular tissue within the cardiomyocyte subcluster 12. Related to Fig. 7e. Statistics are included.

Supplementary Data 5: Top marker genes per cardiomyocyte subcluster. Related to Supplementary Fig. 15.
