# Supplementary material for: Antigen presentation plays positive roles in the regenerative response to cardiac injury in zebrafish
Source: Nat Commun. 2024 Apr 29;15:3637. doi: 10.1038/s41467-024-47430-1 (PMC11058276; doi:10.1038/s41467-024-47430-1)
Supplement: Supplementary file 9 — Reporting Summary [file 41467_2024_47430_MOESM9_ESM.pdf]

nature portfolio

Corresponding author(s): Didier Y. R. Stainier
João Cardeira-da-Silva

Last updated by author(s): Mar 17, 2024

Reporting Summary

Nature Portfolio wishes to improve the reproducibility of the work that we publish. This form provides structure for consistency and transparency in reporting. For further information on Nature Portfolio policies, see our Editorial Policies and the Editorial Policy Checklist.

Statistics

For all statistical analyses, confirm that the following items are present in the figure legend, table legend, main text, or Methods section.

n/a	Confirmed	
☐	☒	The exact sample size (*n*) for each experimental group/condition, given as a discrete number and unit of measurement
☐	☒	A statement on whether measurements were taken from distinct samples or whether the same sample was measured repeatedly
☐	☒	The statistical test(s) used AND whether they are one- or two-sided<br>*Only common tests should be described solely by name; describe more complex techniques in the Methods section.*
☒	☐	A description of all covariates tested
☐	☒	A description of any assumptions or corrections, such as tests of normality and adjustment for multiple comparisons
☐	☒	A full description of the statistical parameters including central tendency (e.g. means) or other basic estimates (e.g. regression coefficient) AND variation (e.g. standard deviation) or associated estimates of uncertainty (e.g. confidence intervals)
☐	☒	For null hypothesis testing, the test statistic (e.g. *F*, *t*, *r*) with confidence intervals, effect sizes, degrees of freedom and *P* value noted<br>*Give P values as exact values whenever suitable.*
☒	☐	For Bayesian analysis, information on the choice of priors and Markov chain Monte Carlo settings
☒	☐	For hierarchical and complex designs, identification of the appropriate level for tests and full reporting of outcomes
☒	☐	Estimates of effect sizes (e.g. Cohen's *d*, Pearson's *r*), indicating how they were calculated

Our web collection on statistics for biologists contains articles on many of the points above.

Software and code

Policy information about availability of computer code

Data collection	Fluorescence images were acquired using ZEN (blue edition) Software.<br>Bright field images were acquired using NIS-Elements 4.30 or Zen Software.<br>RT-qPCR data was obtained using Bio-Rad CFX Manager 3.1 or Thermo Fisher Design & Analysis Software 2.6.0.
Data analysis	Bulk RNA-seq:<br>- Raw reads were assessed for quality, adapter content and duplication rates with FastQC (http://www.bioinformatics.babraham.ac.uk/projects/fastqc).<br>- Reads with a quality drop below a mean of Q15 in a window of 5 nucleotides were trimmed using Trimmomatic version 0.39.<br>- Reads were trimmed, filtered, and aligned against the Ensembl zebrafish genome version danRer11 (Ensembl release 104) using STAR 2.7.9a.<br>- Reads aligning to genes were counted with featureCounts 2.0.2 from the Subread package.<br>- Identification of differentially expressed genes was performed using DESeq2 version 1.30.0.<br>- Dimension reduction analyses (PCA) were performed on regularized log transformed counts using the R packages FactoMineR.<br>- DEGs were submitted to gene set overrepresentation analyses with KOBAS 2.0.<br>- For the generation of heat maps of selected genes, Heatmapper (www.heatmapper.ca; University of Alberta) was used.<br><br>scRNA-seq:<br>- Raw reads were aligned against the zebrafish genome (DanRer11) and counted by StarSolo, followed by secondary analysis in Annotated Data Format.<br>- After preprocessing, cells were analyzed via the Scanpy framework.<br>- Doublets were removed by Scrublet.

- Final data visualization was done using a CellxGene package.

scRNA-seq reclustering analysis of whole zebrafish ventricles:
- Reclustering was performed using Seurat v4

Flow cytometry:
- The flow cytometry results were analyzed using FlowJo v10.8.1

Quantifications:
- Proliferation and cardiomyocyte dedifferentiation were quantified using ZEN (blue edition) software.
- All other quantifications were performed using ImageJ v1.51n.

Data and statistics:
- Data were processed using Prism v9.3.1 and Excel 2016.
- Statistics and graphs were generated using Prism v9.3.1.
- Graphs were edited to their final version using Inkscape 1.2.2.

Sequence analysis:
- Sequence analysis was performed using SnapGene Viewer 5.2.2.

For manuscripts utilizing custom algorithms or software that are central to the research but not yet described in published literature, software must be made available to editors and reviewers. We strongly encourage code deposition in a community repository (e.g. GitHub). See the Nature Portfolio guidelines for submitting code & software for further information.

Data

Policy information about availability of data

All manuscripts must include a data availability statement. This statement should provide the following information, where applicable:

- Accession codes, unique identifiers, or web links for publicly available datasets
- A description of any restrictions on data availability
- For clinical datasets or third party data, please ensure that the statement adheres to our policy

The raw RNA-seq and scRNA-seq datasets have been deposited in the National Center for Biotechnology Information (NCBI) Gene Expression Omnibus (GEO), under accession numbers GSE230669 (https://www.ncbi.nlm.nih.gov/geo/query/acc.cgi?acc=GSE230669) and GSE232061 (https://www.ncbi.nlm.nih.gov/geo/query/acc.cgi?acc=GSE232061), respectively.
Reviewer tokens are avyzsaeahvgzzuh (for GSE230669) and ifizyaumlborxgj (for GSE232061).

Research involving human participants, their data, or biological material

Policy information about studies with human participants or human data. See also policy information about sex, gender (identity/presentation), and sexual orientation and race, ethnicity and racism.

Reporting on sex and gender	N/A
Reporting on race, ethnicity, or other socially relevant groupings	N/A
Population characteristics	N/A
Recruitment	N/A
Ethics oversight	N/A

Note that full information on the approval of the study protocol must also be provided in the manuscript.

Field-specific reporting

Please select the one below that is the best fit for your research. If you are not sure, read the appropriate sections before making your selection.

☒ Life sciences ☐ Behavioural & social sciences ☐ Ecological, evolutionary & environmental sciences

For a reference copy of the document with all sections, see nature.com/documents/nr-reporting-summary-flat.pdf

Life sciences study design

All studies must disclose on these points even when the disclosure is negative.

Sample size	Sample sizes were chosen based on accepted standards in the field using adult animals and previously published literature (such as PMID: 34516874, 26472034 or 29610343). No statistical method was used to predetermine the sample size.

Data exclusions	No data were excluded from analysis.
Replication	All experiments in which statistical significance was necessary were verified with at least 3 biological replicates and/or independent experiments.
Randomization	All experiments used females and males: i) for experiments using an even number of animals, 50% of females and 50% of males were chosen for each group; ii) for experiments using an even number of animals, 50% of females and 50% of males plus one female or one male were used (e.g., if N=3, either two females and one male or two males and one female were used). For experiments using mutants, the genotype was known prior experiments. All animals were chosen randomly considering the above-described conditions.
Blinding	For studies using cd74a; cd74b mutants, wild-type and mutant animals were identified by genotyping before experimental procedures, randomly remixed whenever possible, and re-genotyped at the end of the experiments. Experiments involving the treatment with cyclosporine A were not performed blindly. Image analysis and quantifications were performed blindly.

Reporting for specific materials, systems and methods

We require information from authors about some types of materials, experimental systems and methods used in many studies. Here, indicate whether each material, system or method listed is relevant to your study. If you are not sure if a list item applies to your research, read the appropriate section before selecting a response.

Materials & experimental systems

n/a	Involved in the study	
☐	☒	Antibodies
☒	☐	Eukaryotic cell lines
☒	☐	Palaeontology and archaeology
☐	☒	Animals and other organisms
☒	☐	Clinical data
☒	☐	Dual use research of concern
☒	☐	Plants

Methods

n/a	Involved in the study	
☒	☐	ChIP-seq
☐	☒	Flow cytometry
☒	☐	MRI-based neuroimaging

Antibodies

Antibodies used

Primary antibodies used in this study were:
anti-GFP at 1:200 (chicken; GFP-1010; Aves Labs)
anti-mCherry at 1:200 (chicken; when combined with rabbit anti-Aldh1a2 antibody; CPCA-mCherry; EnCor Biotechnology)
anti-DsRed at 1:100 (rabbit; all the other experiments; recognizing mCherry; Living Colors, 632496; Takara)
anti-Aldh1a2 at 1:50 (mouse; when combined with rabbit anti-pERK or anti-Fli1 antibodies; sc-393204; Santa Cruz Biotechnology, Inc.)
anti-Aldh1a2 at 1:100 (rabbit; all other experiments; GTX124302; GeneTex)
anti-pERK at 1:200 (rabbit; 4370S; Cell Signaling Technology)
anti-Fli1 at 1:100 (rabbit; ab133485; Abcam)
anti-Mef2 at 1:150 (rabbit; DZ01398; Boster Bio)
anti-PCNA at 1:200 (mouse; sc-56; Santa Cruz Biotechnology, Inc.)
anti-Wif1 at 1:100 (rabbit; GTX16429; GeneTex)
N2.261 at 1:20 (mouse; developed by H. M. Blau, obtained from the Developmental Studies Hybridoma Bank, Iowa City, IA, USA).

Alexa Fluor-conjugated antibodies raised in goat (Thermo Fisher Scientific) were used at 1:500.

Validation

All antibodies used in this study were commercially available.

anti-GFP, https://www.aveslabs.com/products/anti-green-fluorescent-protein-antibody-gfp.
Manufacturer: Chickens were immunized with purified recombinant green fluorescent protein (GFP) emulsified in Freund's adjuvant.
Reference: PMID: 34732708

anti-mCherry, https://encorbio.com/product/cpca-mcherry/
Manufacturer: The antibody recognizes mCherry on western blots, in appropriate cells and sections, and does not react with GFP.

anti-DsRed, https://www.takarabio.com/documents/Certificate%20of%20Analysis/632496/632496-101717.pdf
Manufacturer: This antibody recognizes DsRed-Express, DsRed-Express2, mCherry, DsRed2, E2-Crimson, tdTomato, mStrawberry, and mBanana, and both N- and C-terminal fusion proteins containing these fluorescent proteins in mammalian cell lysates.

anti-Aldh1a2, https://www.scbt.com/p/aldh1a2-antibody-g-2
Reference: PMID: 34516874

anti-Aldh1a2, https://www.genetex.com/Product/Detail/Aldh1a2-antibody/GTX124302

Manufacturer: reactivity: zebrafish
Reference: PMID: 36513650

anti-pERK, https://www.cellsignal.com/products/primary-antibodies/phospho-p44-42-mapk-erk1-2-thr202-tyr204-d13-14-4e-xp-rabbit-mab/4370
Manufacturer: reactivity with many species, including zebrafish
Reference: PMID: 35285802

anti-Fli1, https://www.abcam.com/products/primary-antibodies/fli1-antibody-epr4646-ab133485.html
Reference: PMID: 29762122

anti-Mef2, https://www.bosterbio.com/polyclonal-anti-mef2-antibody-dz01398-1-boster.html
Manufacturer: anti-Zebrafish Mef2 Antibody
Reference: PMID: 36513650

anti-PCNA, https://www.scbt.com/p/pcna-antibody-pc10
Reference: PMID: 35264012

anti-Wif1, https://www.genetex.com/Product/Detail/WIF1-antibody/GTX16429
Reference: PMID: 30650349

N2.261, https://dshb.biology.uiowa.edu/N2-261
Reference: PMID: 25557620

Animals and other research organisms

Policy information about studies involving animals; ARRIVE guidelines recommended for reporting animal research, and Sex and Gender in Research

Laboratory animals	Wild-type, transgenic and mutant zebrafish (3 to 12 months of age) used in this study were from the AB strain. The following transgenic and mutant lines were used: Tg(mhc2dab:EGFP)sd6, Tg(ptprc:DsRed)sd3, Tg(mpeg1:EGFP)gl22, Tg(UAS:NTR-mCherry) c264, TgBAC(cd4-1:mCherry)umc13, Tg(gata4:EGFP)ae1, Et(krt4:EGFP)sqet331AEt, Tg(cd74a:Gal4ff; cryaa:mCerulean)bns552, cd74a bns454 and cd74b bns456.
Wild animals	No wild animals were used in this study.
Reporting on sex	All experiments were performed using a ratio of females and males as close to 1:1 as possible. Sex was determined based on standard anatomical procedures described for the species.
Field-collected samples	No field-collected samples were used in this study.
Ethics oversight	All procedures on animals conform to the guidelines from Directive 2010/63/EU of the European Parliament on the protection of animals used for scientific purposes and were approved by the Animal Protection Committee (Tierschutzkommission) of the Regierungspräsidium Darmstadt (references: B2/1218 and B2/1229).

Note that full information on the approval of the study protocol must also be provided in the manuscript.

Plants

Seed stocks	*Report on the source of all seed stocks or other plant material used. If applicable, state the seed stock centre and catalogue number. If plant specimens were collected from the field, describe the collection location, date and sampling procedures.*
Novel plant genotypes	*Describe the methods by which all novel plant genotypes were produced. This includes those generated by transgenic approaches, gene editing, chemical/radiation-based mutagenesis and hybridization. For transgenic lines, describe the transformation method, the number of independent lines analyzed and the generation upon which experiments were performed. For gene-edited lines, describe the editor used, the endogenous sequence targeted for editing, the targeting guide RNA sequence (if applicable) and how the editor was applied.*
Authentication	*Describe any authentication procedures for each seed stock used or novel genotype generated. Describe any experiments used to assess the effect of a mutation and, where applicable, how potential secondary effects (e.g. second site T-DNA insertions, mosiacism, off-target gene editing) were examined.*

Flow Cytometry

Plots

Confirm that:

☒ The axis labels state the marker and fluorochrome used (e.g. CD4-FITC).

☒ The axis scales are clearly visible. Include numbers along axes only for bottom left plot of group (a 'group' is an analysis of identical markers).

☒ All plots are contour plots with outliers or pseudocolor plots.

☒ A numerical value for number of cells or percentage (with statistics) is provided.

Methodology

Sample preparation	Immune cells for scRNA-Seq were isolated from whole ventricles of Tg(mhc2dab:EGFP); Tg(ptprc:DsRed) transgenic reporter zebrafish lines. Ventricles were cut into small pieces and dissociated in a 1× PBS solution containing 200 µg/ml of Liberase DL (Roche) for 20 min at 33ºC with constant agitation and intermittent pipetting every 3 min to promote tissue disaggregation. The cell suspensions were passed through a 35 µm nylon mesh by centrifugation into Falcon round bottom polystyrene test tube (Corning Inc.) at 300 g for 4 min at 4ºC. Samples were placed on ice and a 1:1 volume of ice-cold 20% FBS in PBS solution was added prior to cell sorting. EGFP+ and/or DsRed+ cells were sorted using a FACSAria III (BD) sorter equipped with a 100-µm nozzle.<br>EdCs for RNA extraction and qPCR were isolated from whole ventricles of the uninjured fish or from the border zone and injured tissue cut separately (post-cryoinjury) from ventricles of the Et(krt4:EGFP) zebrafish transgenic line. Tissue dissociation was performed using the Pierce Primary Cardiomyocyte Isolation Kit, (Thermo Fisher Scientific) according to the manufacturer's protocol with few modifications as previously published 99. Cells re-suspended in ice-cold FACS buffer (0.25% BSA in 1X HBSS) were sort purified for the EGFP+ EdCs using the FACSAria III (BD) sorter equipped with a 100-µm nozzle. EdCs were directly collected into ice-cold QIAzol Lysis Reagent (Qiagen) and flash frozen in liquid N2 until RNA extraction.<br>For both immune cells and EdCs, dead cells were excluded using DAPI (Sigma, Cat#D954) using 30mW 405nm excitation paired with 450/50nm band pass filter. EGFP fluorescence was measured with 50mW 488nm excitation paired with 530/30nm band pass filter. DsRed fluorescence was measured with 50mW 561nm excitation paired with 586/15nm band pass filter. As zebrafish samples are highly autofluorescent, and exclusion parameter excited by 50mW 561nm paired with 610/20 band pass filter was used.
Instrument	FACSAria III (BD)
Software	FlowJoTM v10.8.1 software (BD Life Sciences)
Cell population abundance	Sample purity was determined by visualizing the EGFP+ and/ or DsRed positive cell populations alongside the negative cell populations under a fluorescence microscope. Cell abundance differed as per the transgenic lines used and the regenerative time-points that were determined to sort the desired populations.
Gating strategy	Samples were gated by first excluding the debris (SSC-area/FSC-area), followed by doublet exclusion (FSC-area/ FSC-height) and exclusion of DAPI positive dead cells (DAPI-area/FSC-area) to sort the desired cell populations.

☒ Tick this box to confirm that a figure exemplifying the gating strategy is provided in the Supplementary Information.
